# The Elimination of DNA from the Cry Toxin-DNA Complex Is a Necessary Step in the Mode of Action of the Cry8 Toxin

**DOI:** 10.1371/journal.pone.0081335

**Published:** 2013-12-04

**Authors:** Bingjie Ai, Jie Li, Dongmei Feng, Feng Li, Shuyuan Guo

**Affiliations:** School of Life Science, Beijing Institute of Technology, Beijing, China; University of Tennessee, United States of America

## Abstract

Several crystal (Cry) proteins are known to occur as DNA-protein complexes. However, the role of the DNA associated with the activated toxin in the mechanism of action of the Cry toxin has long been ignored. Here, we focused on the DNA-activated Cry toxin complex. Both forms of the Cry8Ca2 and Cry8Ea1 toxins, i.e., with or without bound DNA, were separately obtained. Size-exclusion chromatography analysis indicated that the Cry8Ca2 toxin-DNA complex has a tight or compact structure. The Cry8Ca2 toxin-DNA complex is more likely to move toward the air/water interface and is more hydrophobic than the toxin without DNA. Competitive binding assays indicated that the Cry8Ca2 and Cry8Ea1 toxins without DNA specifically bind to the midgut of *Anomala corpulenta* and *Holotrichia parallela* larvae, respectively. In contrast, the association of DNA with each toxin might result in the nonspecific recognition of the Cry toxin and its target receptor in the insect midgut. The association of the DNA fragment with the Cry8 toxin was shown to protect the Cry protein from digestion by proteases. Based on our results, we propose an additional step in the mechanism of action of the Cry8 toxin and elucidate the function of the associated DNA as well as the importance of the removal of this DNA for the insecticidal activity of the toxin.

## Introduction

*Bacillus thuringiensis* (*Bt*) has been extensively studied because of its ability to produce insecticidal proteins during sporulation. The insecticidal properties of these *Bt* proteins have been commercially exploited as biological insecticides [1], [2]. Many crystal (Cry) proteins exist as protoxins with a molecular mass of 130 kDa. The activation of the protoxin appears to occur via a sequential series of proteolytic cleavages that begin at the C-terminus and proceed toward the N-terminus until a protease-stable toxin, typically having a molecular mass of 65 kDa, is generated [3].

DNA condensation has been observed in the region of crystalline inclusion body formation, which is an important stage for Cry production in *Bt* [4]. It has been reported that some Cry proteins appear to be complexed with DNA [4]. For example, a 20-kbp DNA fragment was observed in Cry proteins isolated from seven subspecies of *Bt* and in the Cry1Aa, Cry1Ab, and Cry1Ac proteins cloned and expressed in *Escherichia coli* [5], [6]. Studies have been conducted to determine the form of DNA associated with the Cry1A protoxin, and only the N-terminal toxic moiety of the protoxin has been found to interact with DNA [5]. Previously, we studied the DNA associated with the activated toxin and speculated that the association of this DNA with the Cry8Ea1 toxin serves to stabilize the protein against aggregation and increases the tendency of the toxin to move toward the phospholipid membrane [6].

The mode of action of the Cry protein involves the following steps: solubilization of the crystals in the midgut, protoxin activation by midgut proteases, binding of the activated toxin to the receptors localized in the apical microvilli of insect midgut cells, oligomerization of the toxin, insertion of the toxin into the apical membrane, and pore formation that ultimately kills the cells [7]. However, the function of the DNA associated with the activated toxin in the mechanism of action of the Cry toxin has not been determined.

Cry8Ca, a type of Cry protein, is toxic to *Anomala corpulenta* and *A. exoleta* larvae, and Cry8Ea is toxic to *Holotrichia parallela* larvae. All of these insect larvae are important pests in agriculture, horticulture, and forestry [1], [8]–[10]. In the present study, the binding specificities of both forms of the Cry8Ea1 and Cry8Ca2 toxins, i.e., with and without bound DNA, to the midgut of the target insect larvae were compared. The Cry8Ca2 and Cry8Ea1 toxin-DNA complex was isolated, and the role of DNA binding was investigated. Based on the these results, we propose an additional step in the mechanism of action of the Cry8 toxin and elucidate the function of the associated DNA as well as the importance of the removal of this DNA for the insecticidal activity of the toxin.

## Materials and Methods

### Materials

The bacterial strain *Bt* HBF-1, which contains the *cry8Ca2* gene (accession No. AY518201) that encodes the Cry8Ca2 protoxin [10], was obtained from the Institute of Plant Protection, Hebei Academy of Agricultural and Forestry Sciences, Baoding, China.

The *Bt* strain HD8E with the plasmid pSTK-8E, which contains the *cry8Ea1* gene (GenBank accession No. AY329081) that encodes the Cry8Ea1 protoxin, was developed and stored at the State Key Laboratory for Biology of Plant Diseases and Insect Pests, Institute of Plant Protection, Chinese Academy of Agricultural Sciences [11], [12].

Superdex 200, Resource-Q, and Sephadex G-50 columns were obtained from Amersham Pharmacia Biotech, and ultra-centrifuge filters were purchased from Millipore. DNase I (RNase-free) was purchased from Takara. CHES and Triton X-100 were obtained from Amresco. Egg-yolk phosphatidyl choline (PC) was purchased from Avanti Polar Lipids (Alabaster, AL), and calcein was obtained from Molecular Probes (Eugene, OR).

Proteinase K, TPCK-treated trypsin, bovine pancreas α-chymotrypsin, cholesterol (Ch), and stearylamine (S) were purchased from Sigma. All other reagents were local products of analytical grade.

### Purification of the Cry8 toxin and Cry8 toxin-DNA complex

The Bt HBF-1 strain was grown at 30°C in PB medium (0.5% peptone and 0.3% beef extract) for approximately 54 h. The Bt HD8E strain was grown, and the protoxin was obtained as previously described [13]. Cell harvesting and crystal purification were performed according to the procedure described by Luo *et al*. [14]. The purification of the Cry8Ca2 toxin, Cry8Ca2 toxin-DNA complex, Cry8Ea1 toxin, and Cry8Ea1 toxin-DNA complex was performed according to a previously described method for Cry8Ea1 [6]. Briefly, the Cry toxin-DNA complex was obtained by initial activation of the protoxin with chymotrypsin followed by purification using size-exclusion chromatography with a Superdex 200 column (HR 10/30) equilibrated with 50 mM Na_2_CO_3_ (pH 10.2) using a Pharmacia FPLC system. The Cry toxin-DNA complex was further treated with DNase I (1 U/mg toxin) at 4°C for 12 h. The Cry toxin without DNA was obtained by a second application to the Superdex 200 column using the same buffer and parameters described above.

### Determination of the surface pressure caused by the penetration of the protein into the air/water interface

The surface pressure of various protein solutions was measured using the Wilhelmy plate method [15] with a NIMA 9000 microbalance (Nima Technology Ltd.; Coventry, UK) as described by Xia and Sui [16]. The Cry8Ca2 toxin or toxin-DNA complex was added to a 50 mM Na_2_CO_3_ (pH 10.2) solution to a final concentration of 0.45 mM. We used this high concentration of toxin to get a surface pressure that keeps almost no significant change while further increase the concentration of the toxin. The change in the surface pressure caused by the penetration of the protein into the air/water interface was measured until a stable reading was obtained.

### Preparation of small unilamellar vesicles (SUVs) and loading of the liposomes with calcein

The SUV preparation was performed as previously described [17]. Briefly, egg-yolk phosphatidyl choline (PC), cholesterol (Ch), and stearylamine (S) were mixed in glass vials in a 7∶1∶2 proportion (µmol), respectively, to a final concentration of 2.6 mol and dried by argon flow evaporation followed by overnight storage under vacuum to remove any residual chloroform. The lipids were hydrated in 2.6 ml of a solution of 10 mM CHES and 150 mM KCl pH 9 for 5 min followed by vortexing. To prepare the SUVs, the lipid suspension was subjected to sonication three times for 2 min using a Branson-1200 bath sonicator. The SUVs were used within 2 – 3 d after their preparation.

Calcein-containing liposomes were prepared by sonication (three times for 2 min) of the SUVs in calcein (80 mM) dissolved in 150 mM KCl and 10 mM CHES, pH 9, as previously described [17]. Non-entrapped calcein was removed by gel filtration on Sephadex G-50 (1×15 cm column) eluted with the same buffer.

### Calcein-release assays

Calcein leakage experiments were performed as previously described [17], [18]. Calcein-loaded SUVs (100 µl) were added to 900 µl of a solution of 150 mM KCl and CHES 10 mM, pH 9, in a glass tube. Calcein fluorescence was excited at 490 nm (2 nm slit) and monitored at 520 nm using a Fluoromax-4 spectrofluorometer (Horiba Jobin Yvon). Finally, different freshly prepared Cry toxins (20 nM) were added to the SUVs and incubated for 20 min. The released calcein induced an increase in fluorescence due to the dequenching of the dye in the external medium. Maximal leakage at the end of each experiment was assessed by lysis with 0.1% Triton X-100 (final concentration). All fluorescence experiments were performed in quadruplicate at 20°C.

### Preparation of brush border membrane vesicles (BBMVs)

The BBMVs were prepared from the midguts of third instar larvae of *A. corpulenta* and *H. parallela* using the differential magnesium precipitation method described by Wolfersberger *et al*. [19]. Leucine aminopeptidase was used as a marker of membrane proteins during BBMV preparation as previously described [20]. The enrichment of Leucine aminopeptidase activity in BBMV preparations ranged from 10 to 15 fold relative to that in crude homogenates. The prepared BBMVs were stored as aliquots at −80°C.

### Competition binding assays

The Cry8Ea1 toxin and toxin-DNA complex were biotinylated using a protein biotinylation kit (Sigma-Aldrich) according to the manufacturer's protocol. The molar ratio in the reaction mixture is ∼13∶1 of biotinylation reagent (BAC-SulfoNHS) to protein.

Briefly, 20 µg of BBMV proteins from *A. corpulenta* or *H. parallela* larvae was incubated with 10 nM biotinylated toxin in the presence or absence of a 50- to 1,000-fold excess of unlabeled toxin in binding buffer (PBS, 0.1% w/v bovine serum albumin, 0.1% v/v Tween 20 at pH 7.6) for 1 h. The unbound toxin was removed by centrifugation for 10 min at 14,000 *g*. The BBMVs were suspended in 100 µl of binding buffer and washed twice with the same buffer. Finally, the BBMVs were suspended in 20 µl of PBS (pH 7.6), and an equal volume of 2× Laemmli sample loading buffer (0.125 M Tris-HCl, pH 6.8, 4% sodium dodecyl sulfate, 20% glycerol, 10% 2-mercaptoethanol, and 0.01% bromophenol blue) was added. The samples were boiled for 5 min, loaded onto SDS-PAGE gels, and electrotransferred to PVDF membranes [21]. The biotinylated protein that remained bound to the vesicles was visualized by incubation with ExtrAvidin-Peroxidase conjugate (1∶10,000 dilution) for 1 h, followed by color development with the ECL Western Blotting Substrate (Pierce), according to the manufacturer's protocol.

### Bioassay

Solutions of Cry8Ea1 toxin or toxin-DNA complex were diluted in 50 mM Na_2_CO_3_ (pH 10.2) as a series of gradient concentrations and then mixed at a ratio of 40∶200 (ml/g) with soil containing 20 15-d larvae of *H. parallela* and their foodstuff (ultraviolet-sterilized potato pieces) as previously described [22]. As the negative control, solutions without the toxins were added to the same soil. All bioassays were conducted at 25°C with a soil humidity of 18 – 20%. Each assay was repeated three times to reduce bias. The larval mortality was scored after incubation for 7 and 14 d to calculate the 50% lethal concentration (LC_50_) of Cry8Ea1 toxin based on probit analysis [23].

### Digestion of the Cry8Ea1 toxin and toxin-DNA complex by Proteinase K

The Cry8E toxin and toxin-DNA complex were digested with different amounts of proteinase K (1∶5, 1∶10, 1∶20, 1∶30, 1∶40 and 1∶50, weight/weight) in 50 mM Na_2_CO_3_ at 37°C for 5 min. Subsequently, phenylmethylsulfonyl fluoride (PMSF) was added to a final concentration of 1 mM to stop the proteolysis. Quantitation of the 65 kDa toxin bands was performed by densitometry analysis using BandScan 4.5 software from Glyko (http://www.glyko.com/BandScan/Features.html).

## Results

### The presence of DNA associated with the Cry8Ca2 protoxin and the activated toxin

**Figure 1:**
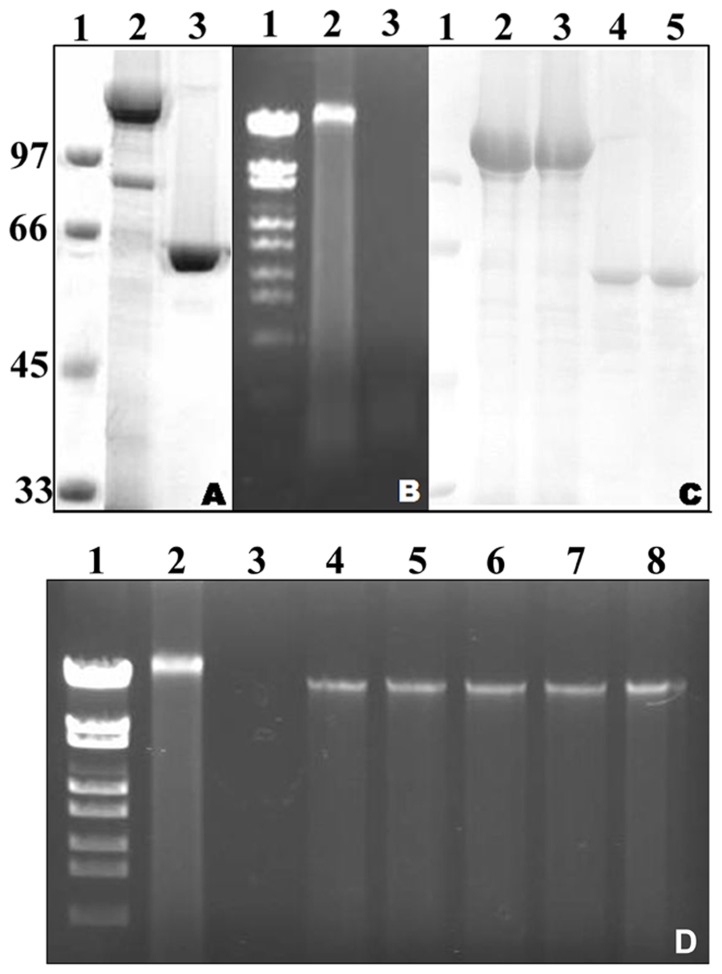
The Cry8Ca2 protoxin-DNA complex. A. Electrophoretic analysis of the Cry8Ca2 protoxin by SDS-PAGE. Lane 1, molecular mass marker; Lane 2, Cry8Ca2 protoxin; and Lane 3, digestion of Cry8Ca2 with chymotrypsin (1∶50, w/w) at 37°C for 1 h. B. The association of DNA with the Cry8Ca2 protoxin. Lane 1, DNA Marker (λ DNA/*Eco*130I: 19329, 7743, 6223, 4254, 3472, 2690, 1882, 1489, 925, and 421 bp); Lane 2, DNA associated with the Cry8Ca2 protoxin; and Lane 3, DNA detection after DNase I treatment at 37°C for 1 h. C. Electrophoretic analysis of the Cry8Ca2 protein by SDS-PAGE after DNase I treatment. Lane 1, molecular mass marker; Lane 2, Cry8Ca2 protoxin; Lane 3, Cry8Ca2 protoxin after DNase I treatment at 37°C for 1 h; Lane 4, further digestion of the Cry8Ca2 protoxin by chymotrypsin (1∶ 50, w/w) at 37°C for 1 h after DNase I treatment; and Lane 5, further digestion of the Cry8Ca2 protoxin by trypsin (1∶50, w/w) at 37°C for 1 h after DNase I treatment. D. Electrophoretic analysis of the Cry8Ca2-DNA complex after DNase I and trypsin digestion. Lanes 1–3 corresponded to lanes 1-3 in B; Lanes 4–5, further digestion of the Cry8Ca2 protoxin by trypsin (1∶30 and 1∶50, w/w) at 37°C for 1 h after DNase I treatment; Lanes 6–7, further digestion of the Cry8Ca2 protoxin by chymotrypsin (1∶30 and 1∶50, w/w) at 37°C for 1 h after DNase I treatment; Lane 8, digestion of the Cry8Ca2 protoxin by proteinase K (final concentration, 50 µg/ml).

The isolated Cry8Ca2 protoxin was analyzed by SDS-PAGE and agarose gel electrophoresis (Fig. 1). Similar to many other Cry proteins, the observed molecular mass of the Cry8Ca2 protoxin was approximately 130 kDa, and it could be activated into the 65-kDa toxin by chymotrypsin (Fig. 1A). An approximately 20-kbp DNA fragment was associated with the protoxin. This DNA fragment appeared to be susceptible to nuclease attack, and digestion with DNase I at 37°C for 1 h eliminated most of the DNA (Fig. 1B). After DNase I digestion, the protoxin could still be activated into the 65-kDa toxin by trypsin or chymotrypsin (Fig. 1C). The initial treatment of the Cry8Ca2 protoxin with DNase I followed by digestion with chymotrypsin or trypsin also resulted in a 20-kbp DNA fragment, which was detected associated with the toxin (Fig. 1D).

**Figure 2:**
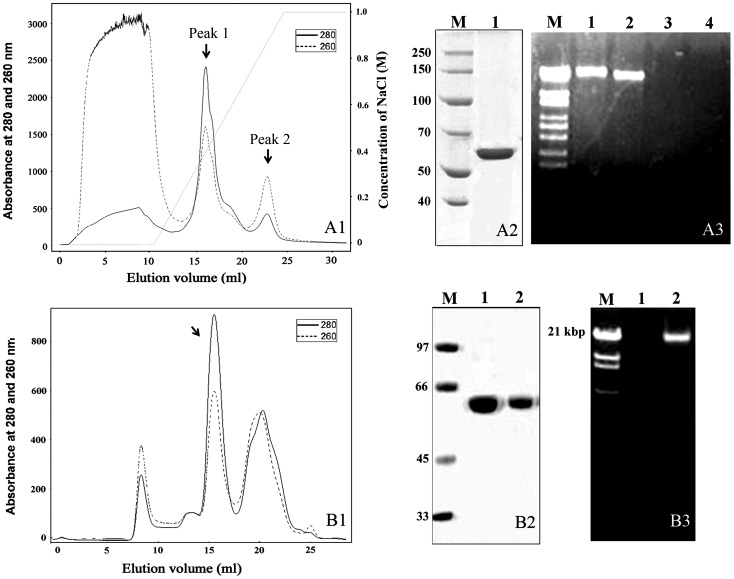
Electrophoretic analysis of the purified Cry8Ca2 toxin and toxin-DNA complex. A. Cry8Ca2 toxin purified using Resource-Q. A1. Elution profile of the Cry8Ca2 toxin. Peak 1 is the fraction containing the Cry8Ca2 toxin. A2. Electrophoretic analysis of the Cry8Ca2 toxin by SDS-PAGE. Lane 1, purified Cry8Ca2 toxin. A3. DNA analysis by 0.7% agarose gel electrophoresis. Lane 1, DNA associated with Cry8Ca2 protoxin. Lane 2, DNA associated with the Cry8Ca2 toxin after chymotrypsin digestion. Lane 3, peak 1 fraction of the Cry8Ca1 toxin. Lane 4, peak 2 fraction of the Cry8Ca1 toxin. B. Cry8Ca2 toxin purified using Superdex 200. B1. Elution profile of the Cry8Ca2 toxin-DNA complex. The peak indicated by the arrow is the fraction containing the Cry8Ca1 toxin-DNA complex. B2. Electrophoretic analysis of the Cry8Ca2 toxin and Cry8Ca2 toxin-DNA by SDS-PAGE. Lane 1, purified Cry8Ca2 toxin (peak fraction of B1 digested with DNase I and collected using Superdex 200) and Lane 2, purified Cry8Ca2 toxin-DNA complex (peak fraction of B1). B3. Analysis of the DNA associated with the Cry8Ca2 toxin and the Cry8Ca2 toxin-DNA complex using 0.7% agarose gel electrophoresis. Lane M, DNA Marker (λ DNA/*Eco*130I); Lane 1, DNA preparation from the Cry8Ca2 toxin; and Lane 2, DNA associated with the Cry8Ca2 toxin-DNA complex.

After activation by chymotrypsin, either ion-exchange chromatography (Resource-Q) or size exclusion chromatography (Superdex 200) was used to obtain the purified Cry8Ca2 toxin (Fig. 2). In ion-exchange chromatography (Fig. 2A1), the Cry8Ca2 toxin was eluted at between 0.2 M – 0.6 M NaCl, as indicated in peak 1 (Fig. 2A2), and was detected without DNA association (Fig. 2A3, Lane 3). Using size exclusion chromatography, the peak fraction indicated by the arrow (Fig. 2B1) was determined to be the fraction containing Cry8Ca2 (Fig. 2B2, Lane 2), which was found to contain bound DNA (Fig. 2B3, Lane 2). After DNase I digestion, the Cry8Ca2 toxin without DNA was obtained after a second application to the Superdex 200 column (Fig. 2B2, lane 1; Fig. 2B3, lane 1).

**Figure 3:**
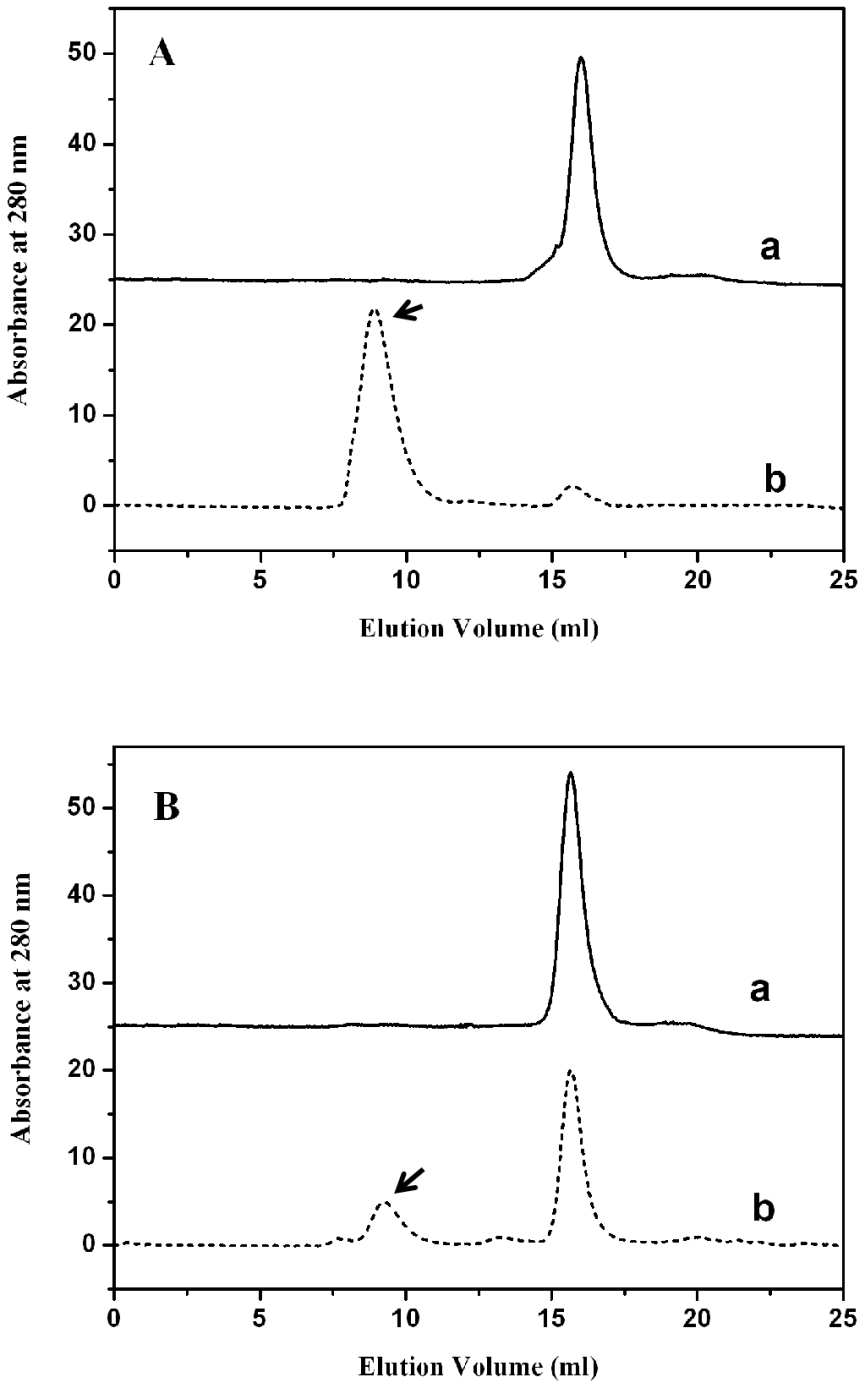
Size exclusion chromatography elution profiles of the Cry8Ca2 toxin and Cry8Ca2 toxin-DNA complex comparing the freshly purified protein with that stored at 4°C for 5 d. A. Elution profiles of the Cry8Ca2 toxin. Profile a is the elution profile of the freshly purified Cry8Ca2 toxin, whereas profile b corresponds to the protein stored at 4°C for 5 d. B. Elution profiles of the Cry8Ca2 toxin-DNA complex. Profile a is the elution profile of the freshly purified Cry8Ca2 toxin-DNA complex, whereas profile b corresponds to that of the protein stored at 4°C for 5 d.

Two aliquots of the Cry8Ca2 toxin, one that was freshly purified and another that had been stored at 4°C for 5 d, were applied to the Superdex 200 column. Identical procedures were performed for the Cry8Ca2 toxin-DNA complex. The elution profiles are shown in Fig. 3. The Cry8Ca2 toxin and toxin-DNA complex had identical elution volumes, indicating that they have a similar molecular size. After storage, most of the Cry8Ca2 toxin aggregated into high molecular weight multimers (the peak indicated by the arrow in Fig. 3A), whereas only a small amount of the Cry8Ca2 toxin-DNA complex had aggregated (the peak indicated by the arrow in Fig. 3B).

### Penetration of the toxin into the air/water interface

**Figure 4:**
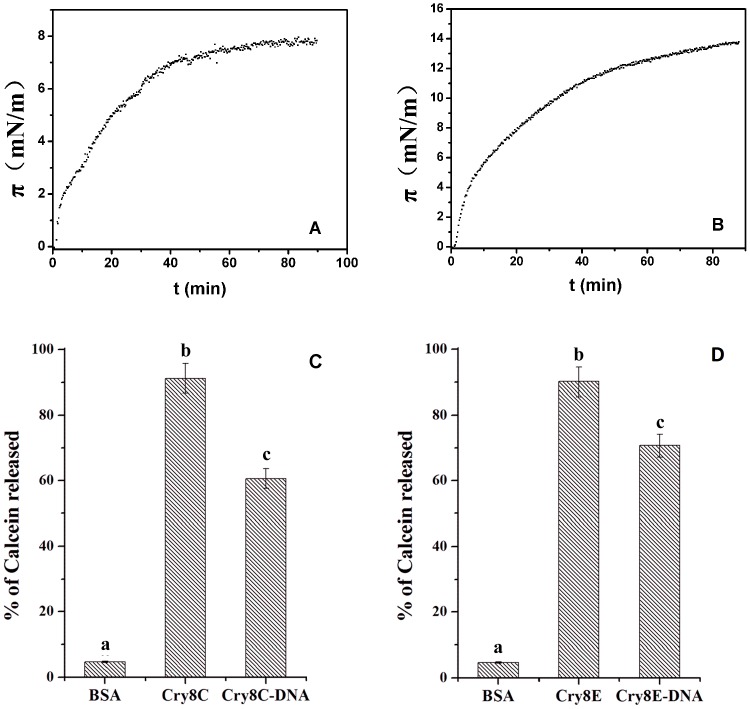
Analyses of the surface pressure caused by the penetration of the protein into the air/water interface and the pore-forming activity based on calcein-release assays. A. The surface pressure of the Cry8Ca2 toxin. B. The surface pressure of the Cry8Ca2 toxin-DNA complex. The final protein concentration was 0.45 mM in a solution of 50 mM Na_2_CO_3_ at pH 10.2. C and D. Analysis of the pore-forming activity using calcein-release assays. Calcein-loaded SUVs were incubated with various protein preparations (final concentration of 20 nM), and the release of calcein was analyzed. The maximal leakage at the end of each experiment was measured by the addition of 0.1% Triton X-100. Each value represents the mean ± SD of four replicate experiments. Bovine serum albumin (BSA) was used as a negative control. A t-test was used to analyze statistical differences of mean values of the percentage of calcein released by the toxin or its toxin-DNA complex when compared with control. Bars labeled with different letters inside each figure indicate that the differences were statistically significant (P<0.001).

The penetration of the Cry8Ca2 toxin and Cry8Ca2 toxin-DNA complex into the air/water interface without the phospholipid was measured. The results (Fig. 4) indicate that the maximum Δπ value induced by the Cry8Ca2 toxin was 7.6 mN/m (Fig. 4A), whereas the maximum Δπ value induced by the Cry8Ca2 toxin-DNA complex was 13.9 mN/m (Fig. 4B). There was about 2 fold difference between the data, which indicate that the Cry8Ca2 toxin-DNA complex is more likely to move toward the air/water interface and is more hydrophobic than the toxin without DNA.

### Pore-forming ability of Cry8 toxin and its toxin-DNA complex analyzed by calcein-release assays

We analyzed the pore-forming ability of the Cry8 toxin and its toxin-DNA complex using SUV liposomes and calcein-release assays. Calcein-containing SUVs were prepared, and the integrity of the lipid membrane vesicles was determined after the addition of the toxins. The release of entrapped calcein from the SUVs was measured as dequenching of the calcein fluorescence and was thereby monitored continuously as an increase in the fluorescence intensity [17]. The data are expressed as a percentage of the maximal fluorescence release obtained using Triton X-100 treatment as the positive control. As shown in Fig. 4 (C and D), the Cry8 toxin and its toxin-DNA complex, including both Cry8E and Cry8C, can cause the release of calcein from the interior of the SUVs. The Cry8E and Cry8C toxins showed a similar extent of calcein release at approximately 90% release, which was greater than that of the toxin-DNA complexes. These findings indicate that both the Cry8 toxin and its toxin-DNA complex were able to form pores and affect the integrity of the SUV liposomes, but that the toxin without DNA showed a stronger ability than did the corresponding toxin-DNA complex. By t-test, it was confirmed that the difference of the pore-forming ability between the Cry8 toxin and its toxin-DNA complex was statistically significant.

### Competitive binding assays

**Figure 5:**
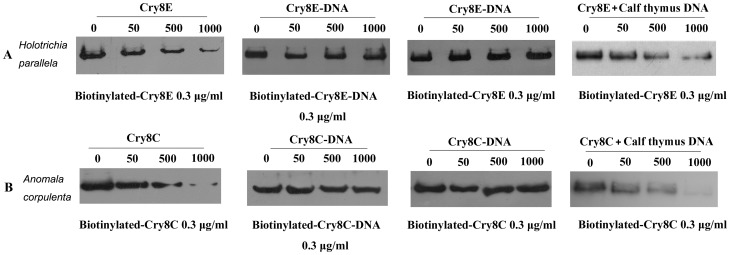
Competitive binding assays of the Cry8 toxin and toxin-DNA complex using the BBMVs from the target larvae. A. Competitive binding of the Cry8Ea1 toxin and Cry8Ea1 toxin-DNA complex with the BBMVs of *H. parallela* larvae. B. Competitive binding of the Cry8Ca2 toxin and Cry8Ca2 toxin-DNA complex with the BBMVs of *A. corpulenta* larvae. Lane 1, homologous competitive binding of the Cry toxin and biotinylated Cry toxin; Lane 2, homologous competitive binding of the Cry toxin-DNA complex and biotinylated Cry toxin-DNA complex; Lane 3, heterologous competitive binding of the Cry toxin-DNA complex and biotinylated Cry toxin; and Lane 4, homologous competitive binding of the Cry toxin and its biotinylated Cry toxin plus calf thymus DNA (0.2 µg in each 100-µl aliquot of competitive binding reaction) as an external DNA control.

The binding of the Cry8Ca2 toxin and toxin-DNA complex to the BBMVs of the *A. corpulenta* larvae in addition to the binding of the Cry8Ea1 toxin and toxin-DNA complex to the BBMVs of the *H. parallela* larvae were analyzed using competitive binding assays. The results of the homologous and heterologous binding of the Cry toxin and toxin-DNA complex to the BBMVs are shown in Fig. 5. In the homologous competitive binding assay, an excess amount of unlabeled Cry8Ea1 toxin or Cry8Ca2 toxin inhibited the binding of the biotinylated Cry toxin to the BBMVs of the target insect. The homologous competitive binding profile indicated that the Cry8Ea1 toxin specifically bound to the BBMVs of the *H. parallela* larvae, whereas the Cry8Ea1 toxin-DNA complex bound nonspecifically to these BBMVs (Fig. 5A). Similarly, the Cry8Ca2 toxin specifically bound to the BBMVs of the *A. corpulenta* larvae, whereas the Cry8Ca2 toxin-DNA complex bound nonspecifically to these vesicles (Fig. 5B). Commercial calf thymus DNA was added to the Cry8E or Cry8C toxin under the homologous competitive binding conditions as a control for external DNA, and the result indicated that external DNA does not interfere with the specific binding. The profile of heterologous competitive binding indicated that the biotinylated Cry toxin-DNA complex does not compete with the Cry toxin while binding to the BBMVs of the insect. This result suggests that the Cry toxin, but not the Cry toxin-DNA complex, specifically recognizes the receptor in the midgut of the target insect.

The LC_50_ of the Cry8Ea1 with or without DNA against the *H. parallela* larvae was determined using laboratory bioassays. The LC_50_ of the Cry8E toxin was 57.50 (25.84 – 341.22) µg/g soil and that of the Cry8E toxin-DNA complex was 47.59 (31.42 – 84.21) µg/g soil. Their 95% fiducial limits partially overlapped.

### Digestion of the toxin-DNA complex by the midgut juices of the target insects and proteinase K

**Figure 6:**
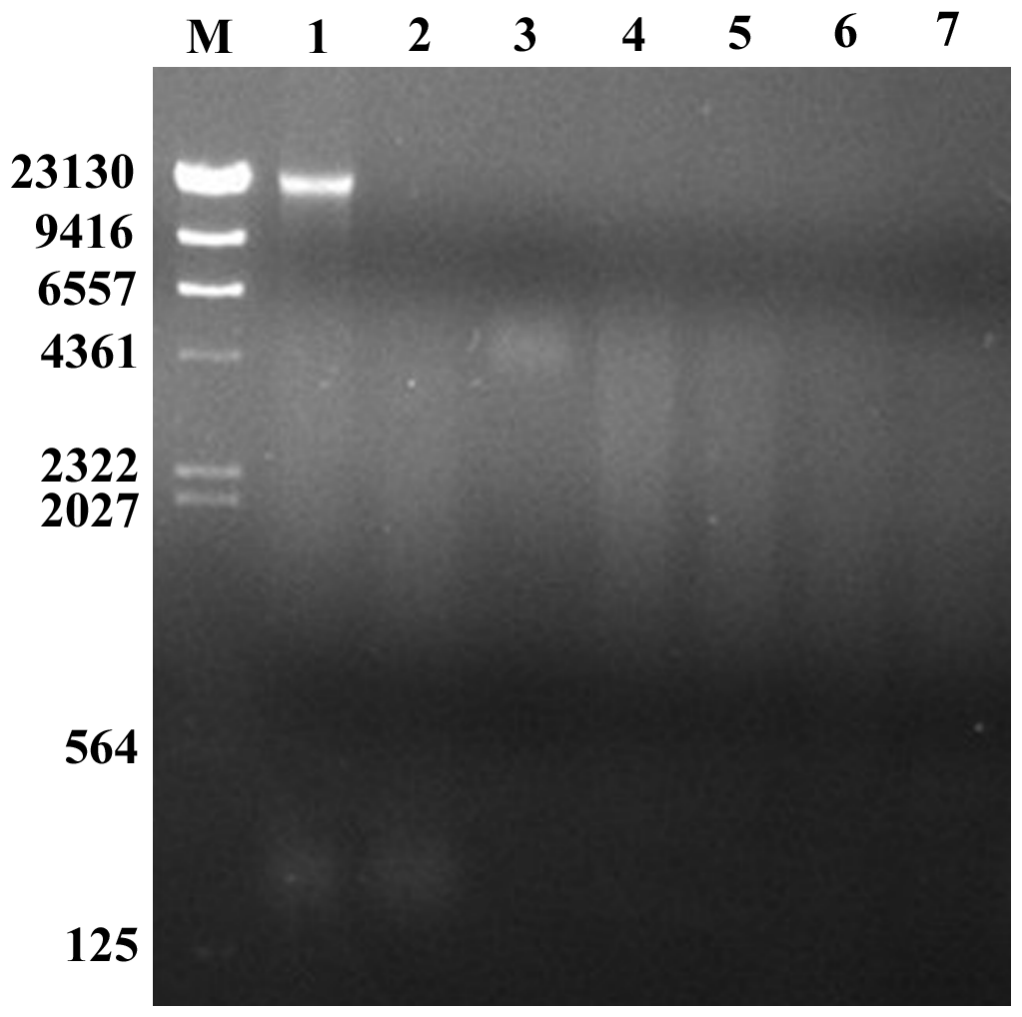
Agarose gel electrophoresis detection of the digestion products of the Cry8E toxin-DNA complex by midgut juice from *H. parallela* larvae. Lane 1, the Cry8E toxin-DNA complex; Lane 2, the Cry8E toxin-DNA complex digested using DNase I at 37°C for 1 h; Lane 3, 0.25% midgut juice; Lanes 4–7, the Cry8E toxin-DNA complex digested using 0.25% midgut juice using 1 µl, 2 µl, 3 µl, and 4 µl, respectively, at 37°C for 1 h.

To determine whether the bound DNA fragment of the Cry8Ea1 toxin can be degraded by the insect, the midgut juice was obtained from *H. parallela* larvae, and the extent of digestion was evaluated. The results showed that the DNA fragment can be degraded by the diluted midgut juice in a manner similar to the degradation observed by commercially available DNase I (Fig. 6).

**Figure 7:**
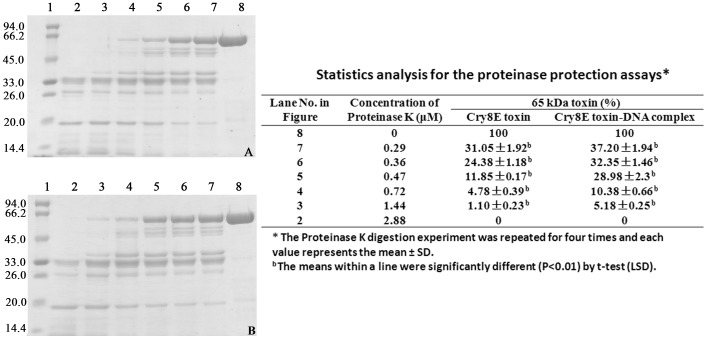
SDS-PAGE analysis of the digestion products of the Cry8E toxin and Cry8E toxin-DNA complex by proteinase K at 37°C for 5 min. A. Digestion of the Cry8E toxin by proteinase K. B. Digestion of the Cry8E toxin-DNA complex by proteinase K. Lane 1, molecular mass marker; Lanes 2–7, products of the digestion of the toxin or toxin-DNA complex by proteinase K at protein (weight/weight) ratios of 1∶5, 1∶10, 1∶20, 1∶30, 1∶40, and 1∶50, respectively; Lane 8A, the Cry8E toxin; and Lane 8B, the Cry8E toxin-DNA complex. A t-test was used to analyze statistical differences of the mean values of the digestion products of the Cry8E toxin-DNA complex by Proteinase K when compared with the Cry8E toxin, as indicated in the right table.

Proteinase K digestion was performed to determine whether the bound DNA of the Cry toxin protects the Cry protein from proteolytic digestion. A titration of the proteinase K concentration was performed in which the Cry8Ea1 toxin and Cry8Ea1 toxin-DNA complex were digested under identical conditions at 37°C for 5 min; the results of this test are shown in Fig. 7. The 65-kDa toxin band was quantified by densitometry analysis, with lane 8 considered to be 100%. As shown in Fig. 7, the residual amount of the 65-kDa toxins under different proteolytic digestion conditions varied with increasing concentrations of proteinase K, and the proteolytic system with the Cry8Ea1 toxin-DNA complex had a higher amount of the 65-kDa toxin remaining relative to the toxin without DNA. The values between them were significantly different (P<0.01) by t-test. This result indicated that the bound DNA fragment of the Cry8Ea1 toxin provides a certain extent of protection of the Cry protein against proteolytic digestion.

## Discussion

Previous studies have shown that the DNA bound to Cry is not an artifact of the crystal purification procedure but is an integral component of the crystal that specifically interacts with the protoxin [4], [5]. Clairmont *et al*. proposed a virus-like structure for the DNA-protoxin complex, described as having a central DNA core surrounded by proteins that interact with the DNA and peripheral ends of the C-terminal region that extend outward [5]. This structure is not supported by the results obtained for Cry8Ea1 and Cry8Ca2 in this present study.

Consistent with the results obtained for Cry8Ea1 [6], the data obtained for Cry8Ca2 indicate that two different DNA fragments were associated with the protoxin: one fragment is susceptible to nuclease attack, likely because it is relatively more exposed, and the second fragment cannot be detected until the protoxin is activated by trypsin or chymotrypsin. The second DNA fragment is associated with the activated toxin. Clairmont *et al*. also demonstrated that protoxin interacts with the DNA via the N-terminal toxic moiety [5]. Our research focused on the DNA-activated toxin complex. The analysis using size exclusion chromatography indicated no obvious differences between the elution volumes of the purified Cry8Ca2 toxin and toxin-DNA complex, which was also observed for the Cry8Ea1 toxin and toxin-DNA complex [6]. Typically, the retention time in size exclusion chromatography should be different for samples that are bound to DNA compared with those free of DNA, especially if the bound molecule is a large DNA fragment. However, the similar elution volumes of the purified Cry8 toxin and Cry8 toxin-DNA indicate that the toxin has a similar size with or without DNA. These results imply that the Cry8 toxin-DNA complex possesses a relatively compact structure compared with Cry8Ea1 and Cry8Ca2. Based on these results, we propose that the structure of the activated toxin may differ before and after the digestion of the bound DNA: when bound to DNA, the activated toxin possesses a tight and compact structure, and upon removal of the constraints imposed by the DNA, the toxin changes to a relaxed structure.

Previously, we proposed that DNA associated with the Cry8Ea1 toxin serves to stabilize the protein from aggregation and increases the tendency of the toxin to move toward the phospholipid membrane [6]. Consistent with the results obtained for the Cry8Ea1 toxin-DNA complex, the Cry8Ca2 toxin-DNA complex is more likely to move toward the air/water interface and is more hydrophobic than the toxin without DNA, indicating that the Cry8Ca2 toxin-DNA complex might increase the tendency of the toxin to move toward the phospholipid membrane. In addition, the results obtained for the Cry8Ca2 toxin-DNA complex confirmed that the associated DNA protects the toxin from nonspecific aggregation. The pore-forming ability of the Cry8E and Cry8C toxins with or without DNA was assessed based on calcein-release assays using SUV liposomes. After removing the associated DNA, both the Cry8E and Cry8C toxins had a stronger pore-forming ability. The associated DNA makes the structure of Cry8 toxin tight and compact, which might influence the pore-forming ability of the toxin. Although the Cry8 toxin-DNA complex is more hydrophobic than the toxin without DNA, the pore-forming ability, whether strong or not, actually is not determined only by its hydrophobic nature.

The interaction of the Cry toxin with different membrane proteins, such as cadherin-like proteins (CAD), aminopeptidase N (APN), and alkaline phosphatase (ALP), is an important process in the mode of action of the Cry toxin [2], [24]. Our results from the competitive binding assays indicated that the association of DNA with the toxin rendered the toxin unable to specifically bind its target receptor from the midgut of the insect. After removal of the DNA from the toxin-DNA complex by DNase I, the Cry8Ea1 toxin specifically bound to its target in the midgut of the insect. Many studies have focused on the interaction of the Cry toxin with insect BBMVs, and most have shown that the Cry toxin interacts with BBMVs specifically, with clear competition of the unlabeled toxin with the toxin-binding sites using standard methods; however, these methods do not account for bound DNA nor make any provision for removing DNA [25]–[31]. In our previous research, no DNA could be detected in the toxin obtained in the main elution peak from the Resource-Q column before or after phenol/chloroform extraction (Fig. 2A). In the other studies, the Cry toxin was purified by anion-exchange chromatography [25]–[31], and thus the obtained Cry toxin might also be lacking DNA.

The bioassay results indicated that the DNA had no obvious influence on the insecticidal activity of Cry8Ea1 toxin. Because we determined that the DNA associated with the Cry8Ea1 toxin can be eliminated by the target midgut juice, the DNA will not interfere with the final specific binding of the toxin with its target in BBMVs.

Our findings support our previous supposition that DNA associated with the *Bt* Cry toxin serves to stabilize the protein from aggregation and increases the tendency of the toxin to move toward the phospholipid membrane. In this work, the presence of a DNA fragment in the Cry8Ea1 toxin-DNA complex was confirmed to protect the Cry protein against digestion by proteases. Furthermore, based on the competitive binding and midgut juice digestion assays, we propose that after the toxin-DNA complex arrives at the BBMVs of the insect, the role of the DNA is fulfilled, and the associated DNA is then digested by the DNases present in the midgut, thereby releasing the toxin from the compact structure and allowing it to be recognized by its receptor. Subsequently, according to the previously reported mechanism of action, the activated toxin binds to the receptors localized in the apical microvilli of the insect midgut cells; after certain conformational changes and oligomerization, the toxin inserts into the membrane to form cation-permeable pores, which cause the swelling and death of epithelial cells by colloid-osmotic lysis [32].

**Figure 8:**
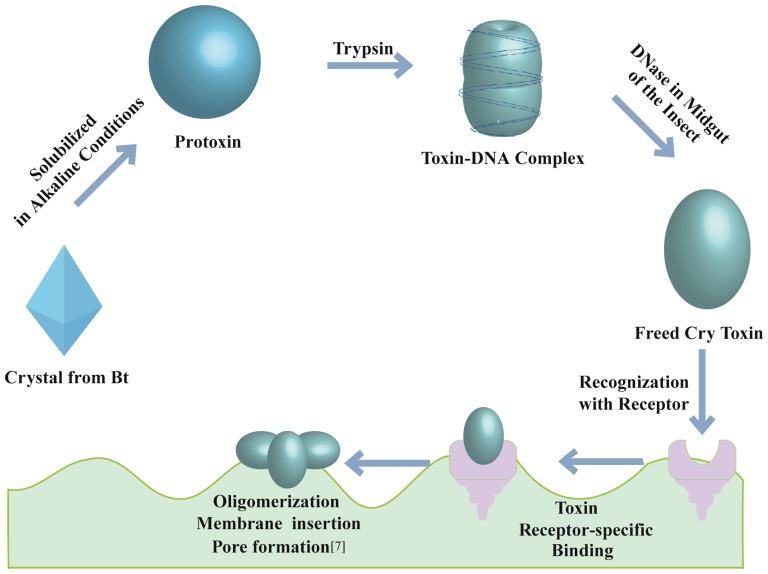
The mechanism of action of the Cry toxin including the proposed role of the toxin-bound DNA.

In conclusion, we propose a supplemental step in the mode of action of the Cry8 toxin (Fig. 8), which emphasizes the presence of DNA associated with the activated toxin and requires the elimination of the DNA for the insecticidal activity of the toxin. Accordingly, variations in the DNase activity in insects may also result in tolerance to the Cry toxin, which requires further investigation. The presence and role of DNA in other Cry toxins should be further analyzed.

## References

[1] Huang DF, Zhang J, Song FP, Lang ZH (2007) Microbial control and biotechnology research on *Bacillus thuringiensis* in China. J Invertebr Pathol 95: 175–180. 17481651 10.1016/j.jip.2007.02.016

[2] Pigott CR, Ellar DJ (2007) Role of receptors in *Bacillus thuringiensis* crystal toxin activity. Microbiol Mol Biol Rev 71: 255–281. 17554045 10.1128/MMBR.00034-06 PMC1899880

[3] Choma CT, Surewicz WK, Carey PR, Pozsgay M, Raynor T, et al (1990) Unusual proteolysis of the protoxin and toxin from *Bacillus thuringiensis*. Structural implications. Eur J Biochem 189: 523–527. 2190826 10.1111/j.1432-1033.1990.tb15518.x

[4] Bietlot HP, Schernthaner JP, Miline RE, Clairmont FR, Bhella RS, et al (1993) Evidence that the CryIA crystal protein from *Bacillus thuringiensis* is associated with DNA. J Biol Chem 268: 8240–8245. 8463332

[5] Clairmont FR, Miline RE, Pham VT, Carriere MB, Kaplan H (1998) Role of DNA in the activation of the Cry1A insecticidal crystal protein from *Bacillus thuringiensis* . J Biol Chem 273: 9292–9296. 9535923 10.1074/jbc.273.15.9292

[6] Guo SY, Li J, Liu YF, Song FP, Zhang J (2011) The role of DNA binding with the Cry8Ea1 toxin of *Bacillus thuringiensis* . FEMS Microbiol Lett 317: 203–210. 21276047 10.1111/j.1574-6968.2011.02230.x

[7] Pardo-López L, Soberon M, Bravo A (2013) *Bacillus thuringiensis* insecticidal three-domain Cry toxins: mode of action, insect resistance and consequences for crop protection. FEMS Microbiol Rev 37: 3–22. 22540421 10.1111/j.1574-6976.2012.00341.x

[8] Ogiwara K, Hori H, Minami M, Takeuchi K, Sato R, et al (1995) Nucleotide sequence of the gene encoding novel delta-endotoxin from *Bacillus thuringiensis* serovar japonensis strain Buibui specific to scarabaeid beetles. Curr Microbiol 30: 227–235. 7765896 10.1007/BF00293638

[9] Sato R, Takeuchi K, Ogiwara K, Minami M, Kaji Y, et al (1994) Cloning, heterologous expression, and localization of a novel crystal protein gene from *Bacillus thuringiensis* serovar japonensis strain buibui toxic to scarabaeid insects. Curr Microbiol 28: 15–19. 7764305 10.1007/BF01575980

[10] Shu CL, Liu RM, Wang RY, Zhang J, Feng SL, et al (2007) Improving toxicity of *Bacillus thuringiensis* strain contains the cry8Ca gene specific to *Anomala corpulenta* larvae. Curr Microbiol 55: 492–496. 17805927 10.1007/s00284-007-9018-3

[11] Shu C, Yan G, Wang R, Zhang J, Feng S, et al (2009) Characterization of a novel cry8 gene specific to Melolonthidae pests: *Holotrichia oblita* and *Holotrichia parallela* . Appl Microbiol Biotechnol 84: 701–707. 19399496 10.1007/s00253-009-1971-2

[12] Shu CL, Yu H, Wang RY, Feng SL, Su XD, et al (2009) Characterization of Two Novel cry8 Genes from *Bacillus thuringiensis* Strain BT185. Curr Microbiol 58: 389–392. 19130127 10.1007/s00284-008-9338-y

[13] Guo SY, Ye S, Liu YF, Wei L, Xue J, et al (2009) Crystal Structure of *Bacillus thuringiensis* Cry8Ea1: An Insecticidal Toxin Toxic to Underground Pests, the Larvae of Holotrichia parallela. J Struct Biol 168: 259–266. 19591941 10.1016/j.jsb.2009.07.004

[14] Luo K, Banks D, Adang MJ (1999) Toxicity, binding, and permeability analyses of four *Bacillus thuringiensis* Cry1 delta-endotoxins using brush border membrane vesicles of Spodoptera exigua and Spodoptera frugiperda. Appl Environ Microbiol 65: 457–464. 9925568 10.1128/aem.65.2.457-464.1999 PMC91047

[15] Demel RA (1974) Monolayers-description of use and interaction. Methods Enzymol 32: 539–544. 4444536 10.1016/0076-6879(74)32052-6

[16] Xia XF, Sui SF (2000) The membrane insertion of trichosanthin is membrane-surface-pH dependent. Biochem J 349 Pt 3: 835–841. 10903146 10.1042/bj3490835 PMC1221212

[17] Rodriguez-Almazan C, Ruiz de Escudero I, Cantón PE, Muñoz-Garay C, Pérez C, et al (2011) The amino- and carboxyl-terminal fragments of the *Bacillus thuringensis* Cyt1Aa toxin have differential roles in toxin oligomerization and pore formation. Biochemistry 50: 388–396. 21142020 10.1021/bi101239r PMC3081932

[18] Rausell C, García-Robles I, Sánchez J, Muñoz-Garay C, Martínez-Ramírez AC, et al (2004) Role of toxin activation on binding and pore formation activity of the *Bacillus thuringiensis* Cry3 toxins in membranes of Leptinotarsa decemlineata (Say). Biochim Biophys Acta 1660: 99–105. 14757225 10.1016/j.bbamem.2003.11.004

[19] Wolfersberger M, Luthy P, Maurer A, Parenti P, Sacchi FV, et al (1987) Preparation and partial characterization of amino acid transporting brush border membrane vesicles from the larval midgut of the cabbage butterfly (Pieris brassicae). Comp Biochem Physiol 86: 301–308.

[20] Garczynski SF, Adang MJ (1995) *Bacillus thuringiensis* Cry1A(c) δ-endotoxin binding aminopeptidase in the *Manduca sexta* midgut has a glycosyl-phosphatidylinositol anchor. Insect Biochem Mol Biol 25: 409–415.

[21] Padilla C, Pardo-López L, de la Riva G, Gómez I, Sánchez J, et al (2006) Role of tryptophan residues in toxicity of Cry1Ab toxin from *Bacillus thuringiensis* . Appl Environ Microbiol 72: 901–907. 16391132 10.1128/AEM.72.1.901-907.2006 PMC1352281

[22] Yu H, Zhang J, Huang DF, Gao JG, Song FP (2006) Characterization of *Bacillus thuringiensis* strain Bt185 toxic to the Asian cockchafer: *Holotrichia parallela* . Curr Microbiol 53(1): 13–17. 16775781 10.1007/s00284-005-0097-8

[23] Finney DJ (1971) Probit analysis. Cambridge University Press, Cambridge, United Kingdom.

[24] Soberón M, Gill SS, Bravo A (2009) Signaling versus punching hole: How do *Bacillus thuringiensis* toxins kill insect midgut cells? Cell Mol Life Sci 66: 1337–1349. 19132293 10.1007/s00018-008-8330-9 PMC11131463

[25] Higuchi M, Haginoya K, Yamazaki T, Miyamoto K, Katagiri T, et al (2007) Binding of *Bacillus thuringiensis* Cry1A toxins to brush border membrane vesicles of midgut from Cry1Ac susceptible and resistant *Plutella xylostella* . Comp Biochem Physiol B Biochem Mol Biol 147: 716–724. 17543562 10.1016/j.cbpb.2007.04.013

[26] Hossain DM, Shitomi Y, Moriyama K, Higuchi M, Hayakawa T, et al (2004) Characterization of a novel plasma membrane protein, expressed in the midgut epithelia of Bombyx mori, that binds to Cry1A toxins. Appl Environ Microbiol 70: 4604–4612. 15294792 10.1128/AEM.70.8.4604-4612.2004 PMC492382

[27] Estela A, Escriche B, Ferré J (2004) Interaction of *Bacillus thuringiensis* toxins with larval midgut binding sites of *Helicoverpa armigera* (Lepidoptera: Noctuidae). Appl Environ Microbiol 70: 1378–1384. 15006756 10.1128/AEM.70.3.1378-1384.2004 PMC368413

[28] Hernández-Rodríguez CS, Van Vliet A, Bautsoens N, Van Rie J, Ferré J (2008) Specific binding of *Bacillus thuringiensis* Cry2A insecticidal proteins to a common site in the midgut of *Helicoverpa* species. Appl Environ Microbiol74: 7654–7659. 10.1128/AEM.01373-08 PMC2607167 18931285

[29] Hua G, Masson L, Jurat-Fuentes JL, Schwab G, Adang MJ (2001) Binding analyses of *Bacillus thuringiensis* Cry delta-endotoxins using brush border membrane vesicles of *Ostrinia nubilalis* . Appl Environ Microbiol 67: 872–879. 11157257 10.1128/AEM.67.2.872-879.2001 PMC92661

[30] Li H, Olson M, Lin G, Hey T, Tan SY, et al (2013) *Bacillus thuringiensis* Cry34Ab1/Cry35Ab1 interactions with western corn rootworm midgut membrane binding sites. PLoS One 8: e53079. 23308139 10.1371/journal.pone.0053079 PMC3537739

[31] Zúñiga-Navarrete F, Gómez I, Peña G, Bravo A, Soberón M (2012) A Tenebrio molitor GPI-anchored alkaline phosphatase is involved in binding of *Bacillus thuringiensis* Cry3Aa to brush border membrane vesicles. Peptides 41: 81–86. 22743140 10.1016/j.peptides.2012.05.019

[32] Schnepf E, Crickmore N, Van Rie J, Lereclus D, Baum J, et al (1998) *Bacillus thuringiensis* and its pesticidal crystal proteins. Microbiol Mol Biol Rev 62: 775–806. 9729609 10.1128/mmbr.62.3.775-806.1998 PMC98934

